# Dwelling Disparities: How Poor Housing Leads to Poor Health

**DOI:** 10.1289/ehp.113-a310

**Published:** 2005-05

**Authors:** Ernie Hood

## Abstract

In recent years, environmental health science has broadened the scope of its inquiries, expanding its investigations beyond the effects of single pollutants on individuals to incorporate the entire panorama of external factors that may affect people’s health. Consideration of the health impacts of the built environment—the human-modified places where we live, work, play, shop, and more—has been a key element in the ongoing evolution of the field of environmental health.

Substantial scientific evidence gained in the past decade has shown that various aspects of the built environment can have profound, directly measurable effects on both physical and mental health outcomes, particularly adding to the burden of illness among ethnic minority populations and low-income communities. Lack of sidewalks, bike paths, and recreational areas in some communities discourages physical activity and contributes to obesity; in those low-income areas that do have such amenities, the threat of crime keeps many people inside. Income segregation—the practice of housing the poor in discrete areas of a city—has also been linked with obesity and adverse mental health outcomes. Lack of a supermarket in a neighborhood limits residents’ access to healthy foods. Dilapidated housing is associated with exposures to lead, asthma triggers (such as mold, moisture, dust mites, and rodents), and mental health stressors such as violence and social isolation.

More recently, the field has begun to take an even wider-angle view, as investigators have begun using innovative new tools and approaches to explore the multifaceted interrelationships between the built environment and population-level health outcomes. It has become increasingly clear that the built environment not only directly impacts our health, but also factors in less direct, complex ways into the health of individuals residing in single-family homes, housing projects, blocks, neighborhoods, and entire cities.

Low-income and/or ethnic minority communities—already burdened with greater rates of disease, limited access to health care, and other health disparities—are also the populations living with the worst built environment conditions. Studies have shown that negative aspects of the built environment tend to interact with and magnify health disparities, compounding already distressing conditions.

Elucidating the associations between the built environment and health disparities has proven to be an enormous challenge to the scientific community, requiring the development of new research paradigms, hypotheses, and methodologies. Traditional studies have often lumped many important components of the built environment into a blanket socioeconomic status variable. But this approach makes it nearly impossible to tease out specific housing and community characteristics related to disease. So, although the traditional tools of environmental health science are still an important part of the mix, research endeavors in this area are now incorporating aspects of sociology, psychology, demography, urban planning, and architecture. Just as significantly, research efforts are reaching beyond the boundaries of the scientific community, embracing rapid translation of research into effective intervention and active collaboration with community members as central concepts in their research protocols.

## Space: The Data Frontier

Spatial analysis is one of the new tools being applied in attempts to quantify the relationship between health disparities and the built environment. One spatial analysis tool, geographic information system (GIS) technology, has long been in use in other areas such as city planning, demography, and epidemiology, but is now allowing environmental and public health researchers to characterize local environments at finely resolved geographic scales. More complete, accurate, and comprehensive geocoded data on resources within communities are becoming increasingly available from a growing number of sources such as governmental agencies, law enforcement agencies, and marketing researchers. This allows researchers studying the built environment to look at communities in new ways.

“The use of GIS technology allows researchers to look at the density and proximity of goods, services, and community resources such as parks, youth clubs, fast food outlets, convenience stores, and other factors that might enhance or hinder health, in relationship to where people live and work,” says Marilyn Winkleby, an associate professor of medicine at the Stanford Prevention Research Center. “GIS provides the technology to spatially display, synthesize, and analyze data—it creates a dynamic visual understanding of people, places, and health.”

As part of a larger project examining neighborhood-level influences on mortality from all causes, Winkleby and her colleagues are preparing to publish results from two studies using GIS to link survey data with other information such as census data, health records, and site visits. Both studies suggest pathways by which disparities in the built environment can be related to disparities in health.

The first study showed that, of 82 neighborhoods studied in four northern/central California cities, the most deprived neighborhoods contained more places that sold alcohol than the least deprived neighborhoods, despite the fact that residents of the higher-SES neighborhoods were the most likely to be heavy drinkers. Winkleby cites this disproportionate clustering in low-income neighborhoods—with its documented sequelae of greater injuries and violence due to increased rates of youth drinking and driving, assault, and car crashes—as one example of why it’s important to examine the built environment in addition to individual risk factors when studying health disparities.

Interestingly, the other study found that another characteristic of the physical environment in the deprived neighborhoods influenced an individual risk factor. “Higher convenience store concentrations—whether measured by density, distance, or number of convenience stores within a one-mile radius of participants’ households—are significantly associated with higher levels of individual smoking,” says Winkleby.

Marie Lynn Miranda, director of the Children’s Environmental Health Initiative (CEHI) at Duke University, says the use of spatial analysis allows her center to more effectively quantify associations between the built environment and health outcomes. “You can create variables of interest and structure them according to what people tell you is the space of interest to them [such as individual homes or unique neighborhoods],” she says. In contrast, earlier studies often used traditional county, zip code, or census tract boundaries, which people often don’t pay attention to as they go about the business of their lives. Customizing study spaces “allows us to create and structure and understand data and influences in a way that is more directly linked with how people live their lives,” Miranda says.

Miranda’s research focuses on identifying interventions that will prevent harmful environmental exposures in children, rather than mitigating existing exposures that may have already affected children. Among several CEHI programs currently in progress, the farthest along is an initiative to prevent childhood lead exposure called Mapping for Prevention. The group has created GIS-based lead exposure maps for 36 North Carolina counties and several other sites around the country using census data, blood lead screening data, and county tax assessor data to identify high-risk areas for lead poisoning. “The notion here is to try to figure out the places that are likely to [contribute] to elevated blood lead levels in children,” says Miranda, “and go in there and do something about the housing stock before a child gets lead-poisoned.”

Once an exposure map has been developed, the researchers work with local health departments to selectively and proactively screen children for lead exposure, and to educate new parents who live in high-risk housing about how to prevent their children from being exposed. They also help housing departments identify ways to prioritize housing rehabilitation and lead abatement funding to address the housing at greatest risk for lead poisoning.

Miranda is convinced that the spatial component is vital: “The work we’re doing at this very highly resolved geographic scale is identifying the risk levels at the individual tax parcel unit, and it provides us with absolutely the most powerful tool to move from mitigation to prevention in childhood lead exposure.” [For more information on GIS projects at Duke, see “Mapping the Air in Public Schools,” p. A308 this issue.]

## Drawing on Community Knowledge

One of the most significant recent developments in efforts to characterize and ameliorate built environment conditions associated with health disparities has been the growing movement toward community-based participatory research (CBPR), which has been largely pioneered and supported by the NIEHS through a variety of extramural grant programs. CBPR studies focus on gathering and disseminating scientific knowledge about the interrelationships between the physical and social environments and health, and to identify, evaluate, and implement potential interventions—all with a distinct emphasis on active collaboration with residents and other stakeholders within the communities being studied. These multidisciplinary projects generally draw from many resources to arrive at comprehensive understanding of the multifaceted dynamics at work in populations suffering health outcome disparities—including negative aspects of the built environment.

Detroit’s Healthy Environments Partnership is an example of a CBPR endeavor. Academic researchers from the University of Michigan are working directly with several community groups and health service providers to examine the contributions of the physical and social environments to both ethnic and socioeconomic disparities in risk factors for cardiovascular disease among the adult population of Detroit. Part of the partnership’s wide-ranging data collection involves assessing aspects of the built environment: housing conditions, sidewalk conditions, land use, concentrations of airborne particulate matter, and access to grocery stores, parks, and recreation areas.

Data have been gathered in three demographically diverse Detroit neighborhoods that were initially selected because they were anticipated to vary in their concentrations of airborne particulate matter. Air quality monitoring confirmed this differential exposure. The researchers also surveyed neighborhood residents, collected blood and saliva samples to assess physiological indicators of cardiovascular risk and stress, and sent observers into the neighborhoods to evaluate the built and social environments in each area.

Although the group is still analyzing the data, principal investigator Amy Schulz, a research associate professor of health behavior and health education at the University of Michigan School of Public Health, says they are seeing trends suggestive of variations in both cardiovascular disease risk factors and protective factors that play out for different racial and socioeconomic groups across areas of the city. For example, they have noted variations in dietary intakes of fruits and vegetables in population groups within the city, and intend to analyze whether conditions in the built environments of the neighborhoods can predict those variations.

Residents of the Detroit neighborhoods being studied have been involved at all stages of the project, including helping to design the survey as well as collect and analyze the data. “They are extremely knowledgeable about the research that has been conducted,” says Schulz, “and understand quite well the results that we are seeing and their implications for the community, because they have been so integrally involved every step of the way.”

Also, says Schulz, community involvement contributes to a very rich analysis whose results are more likely to lead to change. “It’s really important for us to have good information about the impact of the built environment and the social environment on health, but it’s clear that information alone is not going to create change,” she says. “Engaging community members in processes begins to build community mobilization efforts for change.”

Positive changes to the built environment that help reduce health disparities can and do emerge from these partnerships. One such success story is San Diego’s Environmental Health Coalition (EHC), a 25-year-old nonprofit organization, funded largely by a grant from the U.S. Department of Housing and Urban Development (HUD) through the Community Environmental Health Resource Center. This Washington, DC, group helps community organizations develop their capacity to document environmental health hazards in substandard housing and to pursue effective organizing and advocacy strategies for corrective and preventive action [for more information, see “Community Environmental Health Resource Center,” p. A303 this issue]. The EHC works with university researchers, government agencies, and community members to address toxic pollution, land use issues, and substandard housing conditions in San Diego’s low-income communities of color.

Much of that effort has focused on the Barrio Logan, a low-income Latino community plagued by poor air quality due to heavy diesel truck traffic from a major regional freeway dissecting the community, and a troubling mix of industries and residences in close proximity to one another. Armed with results of an informal community health survey that showed disturbing levels of asthma and other respiratory problems, the group convinced the California Air Resources Board to begin monitoring air quality in the community. Since then, says EHC research director Joy Williams, “We’ve gotten the city of San Diego to agree to reroute truck traffic around the community and not through it—one solution to the problem of diesel exhaust in the community. EHC and the community are also working on changes to land use and zoning that will reduce the number of warehouses and industries that generate diesel truck traffic in residential areas.”

The air monitoring also led to a direct intervention to help a family in distress. The group had identified a metal-plating shop on a residential street in Barrio Logan as a potential hazard, and asked for air samples to be collected in the immediate vicinity. Regulators previously had believed that an operation of that size that was basically in compliance would not pose much of a hazard. However, once they sampled the air, they found high levels of emissions such as chromium-6, a highly toxic air pollutant, at the houses next door and across the street, says Williams. It turned out that one family living next door had a son with poorly controlled asthma. Ultimately, the metal-plating shop was forced to close (although many businesses in similar circumstances simply relocate), reportedly leading directly to dramatic improvements in the boy’s health. The EHC continues to make such unhealthful mixed land uses a priority in its activities.

## Housing Developments

Housing is perhaps the ultimate nexus between the built environment and health disparities, and it has been the focus of much recent research and intervention activity looking at new approaches to old problems. The intimate connection between housing and health has been well known for more than a century—Florence Nightingale once wrote, “The connection between health and the dwelling of the population is one of the most important that exists.” But today there is renewed interest in discovering the complex pathways connecting housing factors, neighborhood factors, social factors, adverse health outcomes, and disproportionate disease burden in poor and ethnic minority communities—particularly with respect to skyrocketing rates of chronic diseases such as asthma, obesity, and diabetes.

That renewed interest is being manifested at the national and international levels, as well as in the form of grassroots community action. In late 2004, the World Health Organization convened its 2nd International Housing and Health Symposium at Vilnius, Lithuania, a conference designed to review the existing scientific evidence on housing and health relationships, and assess needs for further research. In what may prove to be a development with wide-ranging global impact, the symposium generated the Vilnius Declaration, in which 250 scientists and officials representing 24 countries committed themselves “to taking action to ensure that health and environmental dimensions are placed at the core of all housing policies (from housing construction and rehabilitation plans, programmes and policies to the use of adequate building materials) and that healthy conditions are ensured and maintained in the existing housing stock.”

In January 2005, indoor environmental quality took center stage at the Surgeon General’s Workshop on Healthy Indoor Environment. The two-day gathering of more than 300 experts from government, academia, the building sciences industry, and public interest groups focused on increasing attention to the issue of indoor air pollution, with the surgeon general and other participants calling for action to improve the health of Americans by improving indoor environments.

At the local level, two of the many CBPR projects in progress around the nation demonstrate the multifaceted, collaborative approach being taken toward not only characterizing housing and health pathways, but designing, implementing, and evaluating interventions as well. Both have come about in response to the high prevalence of asthma in low-income urban communities, with a special focus on improving the health and housing conditions of public housing residents.

Boston’s Healthy Public Housing Initiative (HPHI) is a collaboration among public housing tenants’ right groups, the Committee for Boston Public Housing, the Peregrine Energy Group, and several area universities and city agencies. The collaboration has produced guidance for builders, architects, and others on ways to make both new construction and existing housing healthier. Participants are currently engaged in a four-year project to assess the effectiveness of HPHI asthma intervention programs in three public housing developments. The interventions include installation of air filters, purchase of new mattresses, heavy-duty cleaning, integrated pest management, family education on controlling asthma triggers, and installation of building systems upgrades and modifications.

The group published a study in the 7 December 2004 edition of the online journal *Environmental Health: A Global Access Science Source* reporting the results of a detailed baseline evaluation of 78 asthmatic children living in the three public housing developments. Among other findings, the study showed that many of the children, although they had access to primary care physicians, were not receiving care according to professional asthma management guidelines, which include recommended medications, monitoring practices and equipment, and other measures. Also, exposure to violence, which has been related to exacerbation of asthma symptoms, was a significant problem. In one development where a series of murders had taken place during the study period, 60% of the children were never allowed outside to play.

Add those factors to substandard housing, high concentrations of local ambient air pollution, and other negative aspects of neighborhood built environments, and it starts to become clear why the prevalence and incidence of asthma has risen so sharply and disproportionately among low-income minority urban children. The study report concluded, “Given the elevated prevalence of multiple risk factors, coordinated improvements in the social environment, the built environment, and in medical management would likely yield the greatest health benefits in this high-risk population.”

A CBPR project currently under way in Seattle known as the High Point Healthy Homes and Community Project is taking full advantage of a unique opportunity to simultaneously address built environment issues in the public housing context and gain useful knowledge about how comprehensive interventions can be used to improve the health and well-being of residents. The Seattle Housing Authority is in the process of reconstructing its public housing stock, to replace old, deteriorating structures with town homes. One of the sites being updated is High Point, formerly a 716-unit development, which is now being rebuilt as a 1,600-unit mixed-income community.

The High Point Healthy Homes and Community Project is taking a multilevel approach to designing a public housing development to be a healthy, sustainable community. Developers are thoughtfully addressing a range of considerations, from design issues such as layout, walkability, and watershed protection, to the use of construction materials and practices that enhance indoor environmental quality. This project of the local public health department, housing authority, social service providers, public housing residents, and the University of Washington, with funding from the NIEHS and HUD, is paying particular attention to the needs of families affected by asthma.

“At the individual housing unit level, we made an estimate of the number of families living in High Point affected by asthma, and are now building specially enhanced units which will minimize exposure to asthma triggers by improving the indoor environmental quality,” says James Krieger, principal investigator on the NIEHS-funded component of the project, who is an epidemiologist with the Seattle–King County Department of Public Health and a clinical associate professor of medicine and health services at the University of Washington. (Tim Takaro, also at the University of Washington, led the HUD-funded component.) Thirty-five “Breathe Easy” demonstration homes will feature hardwood flooring (instead of carpeting, which can outgas and cause respiratory problems) and enhanced ventilation systems, weatherization, and insulation to minimize humidity and moisture intrusion, costing an additional 3–4%, or roughly $5,000, more than the development’s standard units (which will also go well beyond building code requirements in several specifications).

Researchers will follow the families for a year before they move into the units to establish a baseline assessment of their asthma status, and then continue to follow them for a year after they move into their new homes, which are currently under construction (the first families are scheduled to move in in fall 2006). “This will be one of the first studies designed as an intervention of people moving into [better]-quality housing while having a health outcome that’s clearly measurable like asthma, to see what the health impact is,” says Krieger.

The Seattle Housing Authority and its organizational partner, Neighborhood House, have also actively sought residents’ input in the design of the new community. It is slated to include walking paths and trails, mini-parks and one larger park, a grocery store, a public library, and a community health center, in hopes that these amenities will provide a built environment more conducive to health and social interaction. A community-based education initiative is also under way, using trained teams of community residents called project action teams to teach their neighbors about basic principles of how to keep their homes and community healthy.

Krieger says they will conduct before-and-after community surveys assessing people’s physical activity, social cohesion, and other factors. “Hopefully, we’ll be able to empirically test the whole notion that a change in the built environment will change health behaviors and increase community cohesion and social capital,” he says. “There’s limited empiric data out there on that now.” [For information on another innovative Seattle housing project, see “Growing Green Communities,” p. A300 this issue.]

David Jacobs, a HUD housing expert, sees wide-ranging potential in projects that quantify such benefits. “If we are able to fully value those types of investments in houses that produce positive health outcomes,” he says, “then we can end the cost-shifting that causes both higher medical bills and higher housing costs. Right now, the benefits of health investments in housing or communities are largely hidden, with avoidable—and usually much higher—costs being absorbed by the medical care sector, after the harm has already been done.”

## The Search for Solutions, Large and Small

Public health and urban planning, sectors that once were closely aligned, have drifted apart over the decades, evolving into professional specialties with too few opportunities to collaborate and little mutual influence, some say. Many practitioners seeking solutions to the festering problems of health disparities and built environment inequities see reconnection of the two fields as a critical goal.

Jason Corburn, codirector of the Center for Occupational and Environmental Health at Hunter College of the City University of New York, strongly advocates recoupling the fields so as to build health considerations into land use, zoning, community design, and other urban planning decisions that to a large extent shape the long-term nature of the built environment. “Too often we’re quantifying housing quality or some aspect of the built environment,” says Corburn, “but we’re not looking historically and at the present time at how these urban planning and land use decisions are being made—who’s involved, what are the processes, who has access to the information. Without that kind of political analysis of the public decision making behind it, I think we’re missing a big piece of the built environment–health disparities puzzle.”

Corburn has helped initiate and manage the development of a health impact assessment process in San Francisco, which is rezoning several neighborhoods in its Mission District. Corburn is working with the city’s public health department and a group of nearly 40 stakeholders including tenants’ rights organizations, business owners, and other community organizations. Their mission is first to define the community’s ideas of the physical and social characteristics of a healthy neighborhood, and then to construct a plan to incorporate those elements as distinct goals of the rezoning process. “The health impact assessment process in San Francisco is an experiment,” Corburn says, “but I think it’s very promising. This kind of approach holds the potential to reconstruct the boundaries of environmental health.”

Prevention Institute, a nonprofit group based in Oakland, California, works with governments, communities, and organizations to establish prevention-oriented health programs and policies. This institute is actively promoting increased participation by public health practitioners in the built environment decision-making process. To help foster greater understanding of the potential role public health can play in improving health outcomes and reducing disparities by altering the built environment, the group recently released a report, *The Built Environment and Health: 11 Profiles of Neighborhood Transformation*, that highlights local success stories across the country. The profiles include inner-city yard lead abatement efforts in Boston, separate successful drives to close nuisance liquor stores in south Los Angeles and open a grocery store in a deprived community in Rochester, New York, and programs that have brought amenities such as jogging paths, bike and walking trails, mural arts, and community gardens to other municipalities. All of the interventions have involved collaborations among community groups, local governments, and public health officials, and have been aimed at reducing health disparities and improving the health of residents of low-income communities.

One of the Prevention Institute’s projects to help communities address health disparities is called THRIVE (Community Tool for Health and Resilience in Vulnerable Environments). THRIVE is a toolkit designed to aid communities in comprehensively assessing their conditions along a scale of risk to resiliency. Executive director Larry Cohen illustrates the concept: “When we think about risk, a street that does not have sidewalks or a separate place for bicyclists is going to be far more dangerous than what we call a ‘complete street,’ which functions for public transportation, for automobiles, for pedestrians, and for bicyclists. Obviously, the first street is going to promote risk, with far more likelihood of automobile crashes and air quality health-related issues, and the second is going to promote resiliency, with more opportunities for physical activity. So a risk can be ameliorated and create a community which will be more resilient.”

The THRIVE toolkit describes 20 community factors within four interrelated clusters: built environment, social capital (which includes societal factors such as social cohesion and trust, civic engagement and participation, and broadly shared beliefs and standards of behavior), services and institutions, and structural factors, with a depiction of risk and resilience for each factor. “This is about looking broadly at the community and creating environments in communities where people want to live, where they can be healthy and safe,” says program manager Manal Aboelata. This can have tremendous impact on not just physical health but also mental health. The toolkit has been successfully pilot-tested in both rural and urban communities, and is currently awaiting final approval from federal funding agencies prior to being available nationally.

## A Concrete Future?

Can this new paradigm within the environmental health sciences—with its multi-disciplinary, systems-level approach to interactions between the built environment and health disparities—actually help to solve some of these long-standing, large-scale problems? Even with the latest scientific knowledge and the enthusiastic participation of a multiplicity of stakeholders, can profound, lasting change realistically be expected to follow? Although there are many encouraging trends, the challenges are formidable and complex, and observers active in the field understand that their campaign will be long, with no assurance of victory.

Carlos Mendes de Leon, an epidemiologist at Rush University Medical Center in Chicago, is investigating the biological and environmental mechanisms by which socioeconomic deprivation leads to disability in older people. His comments on the potential for broad improvements at the societal scale are representative of those heard from many researchers who are determined to soldier on: “At this moment, it’s hard to see this kind of knowledge and understanding of the built environment and health disparities having very concrete ramifications for public policy and medical interventions,” he says.

“But at the same time,” he adds, “we still have to build up the knowledge base so that when conditions change in the broader political sphere, we can point to concrete evidence and concrete strategies that may make an effect.”

Yet there have been many important achievements that demonstrate the potential power of these ideas. For example, the number of both lead-poisoned children and houses with lead-based paint have been reduced over the past decade thanks to concerted action by policy makers and community members. Many diseases that still plague the developing world, such as typhoid and cholera, have been largely eradicated in the United States due in part to improvements in housing density, ventilation, and reliable community water supplies. Learning from these examples should be a first step toward restoring the connections between environmental health, housing, urban planning, and the built environment so that many of the diseases that still plague us today can also be wiped from our lives.

## Figures and Tables

**Figure:**
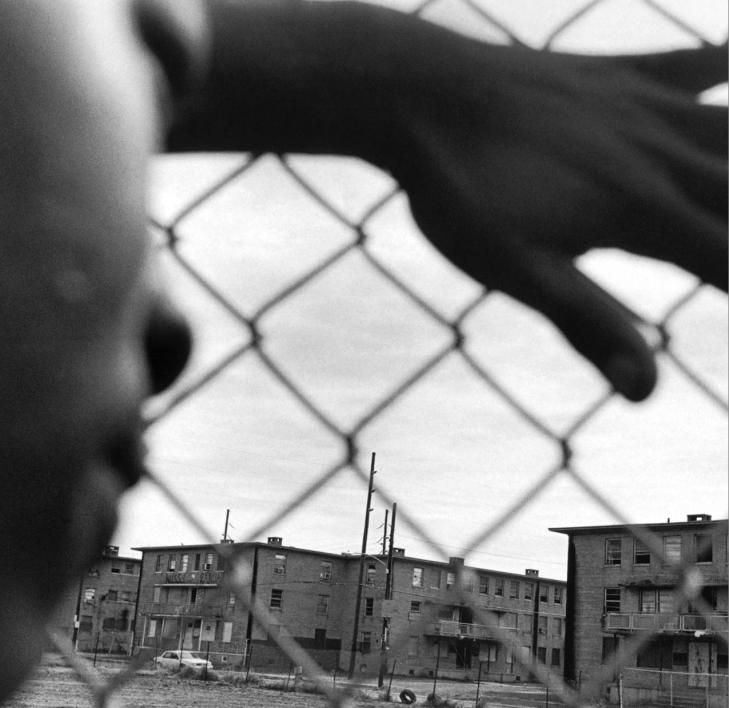


**Figure:**
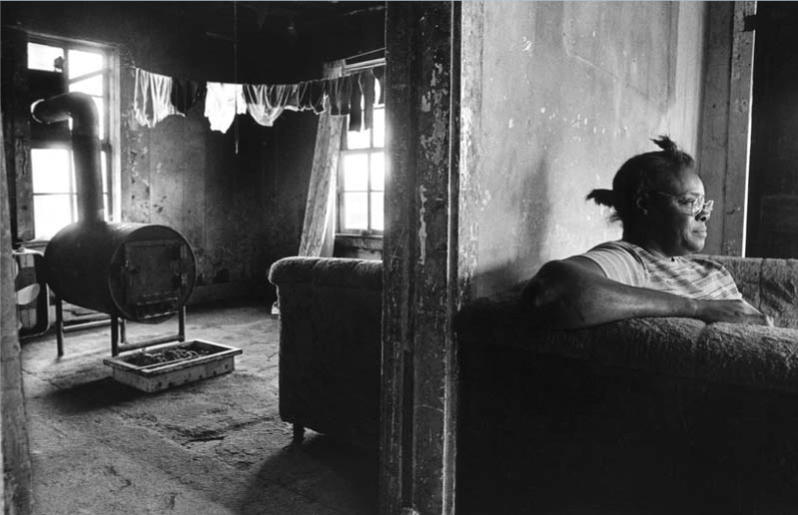
**Home, bitter home?** Tradition holds that home is a haven, where people are protected and nurtured. For many, however, home is a health hazard when factors such as poverty, environmental contamination, and poor design combine to cause or exacerbate disease.

**Figure:**
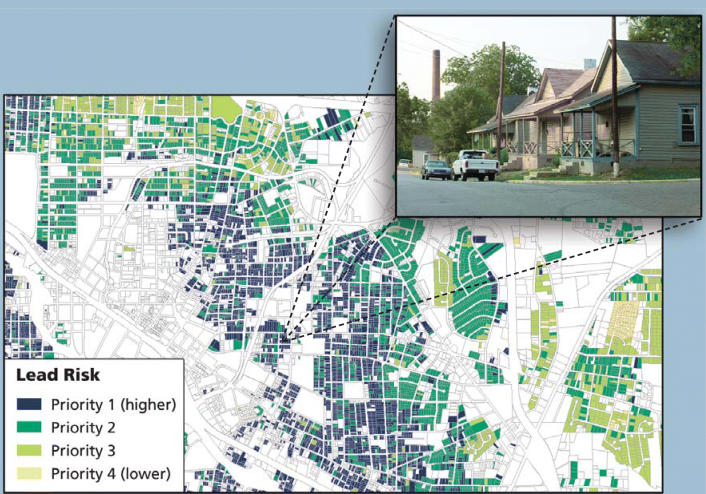
**New tech on the block.** GIS technology can help public health officials pinpoint the worst environmental health hazards on a house-by-house basis. Here, researchers mapped expected lead exposures in Durham, North Carolina.

**Figure:**
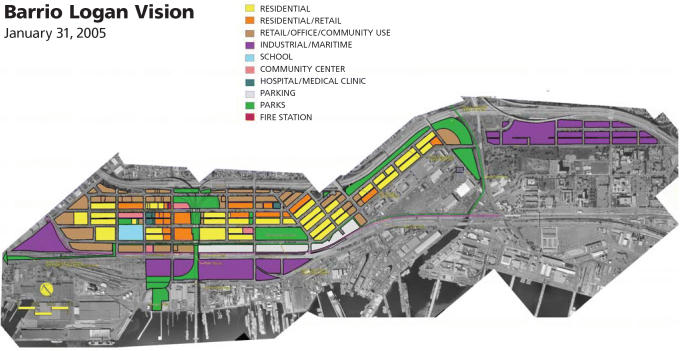
**Envisioning a healthier community.** The Environmental Health Coalition is working with Barrio Logan, a low-income Latino community in San Diego, California, to map out a plan for future land uses to help eradicate hazards and improve health.

**Figure:**
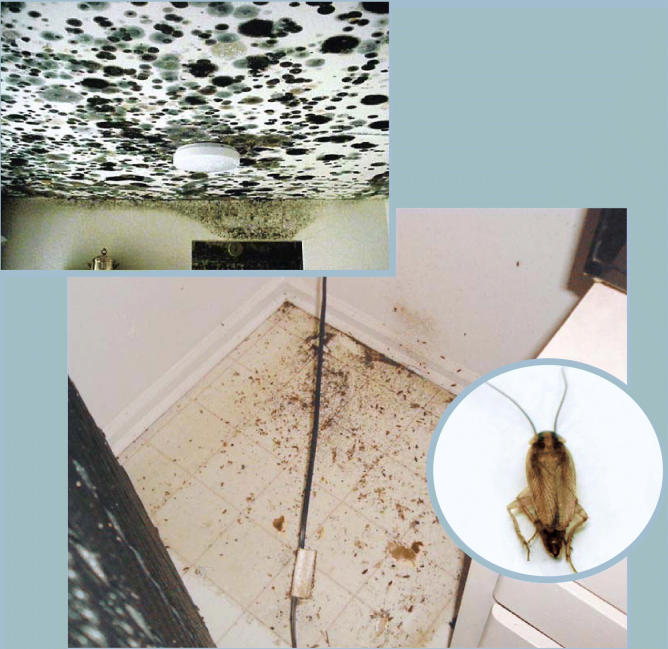
**It came from within.** Some of the worst home health hazards, such as the black mold crawling across the ceiling of an apartment (left) and the cockroach droppings blanketing the floor behind a refrigerator (below), arise inside homes that are poorly maintained or designed. Indoor mold and cockroach antigens have both been associated with worsened asthma and other adverse health effects.

**Figure:**
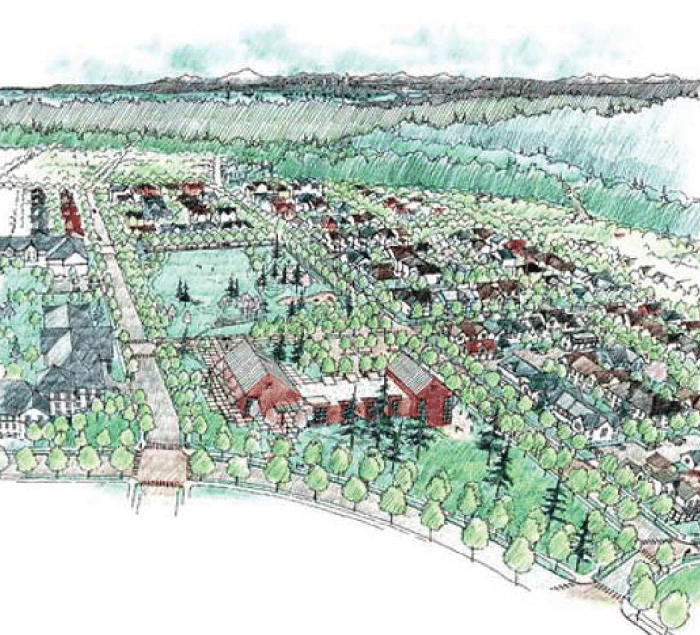
**Making houses into healthy homes.** The High Point Healthy Homes and Community Project in Seattle is employing a new paradigm for public housing: design with health and sustainability in mind.

